# Community‐Based Interventions Improve the Quality of Life for Children Living With Disabilities, Zambia

**DOI:** 10.1111/cch.70203

**Published:** 2026-01-07

**Authors:** Mezan Ghebre, Renee Hepperlen, Jennifer Biggs, Edgar Lunda, Watson Mwandileya, Paula Rabaey, Mary O. Hearst

**Affiliations:** ^1^ Division of Epidemiology and Community Health University of Minnesota Minneapolis Minnesota USA; ^2^ School of Health St. Thomas University St. Paul Minnesota USA; ^3^ Henrietta Schmoll School of Health St. Catherine University St. Paul Minnesota USA; ^4^ Access to Health Zambia Lusaka Zambia; ^5^ Occupational Therapy Department University of Minnesota Minneapolis Minnesota USA; ^6^ School of Nursing University of Minnesota Minneapolis Minnesota USA

**Keywords:** children living with disabilities, community‐based programme, quality of life, Zambia

## Abstract

**Background:**

Children living with disabilities in Zambia face many barriers to quality of life. This study evaluated any participation in activities, the number of activities and which activities of a community‐based intervention impacted quality of life among children living with disabilities and their families.

**Methods:**

We used a pre–post evaluation design for 228 families with a child living with disabilities who participated from 2019 to 2021 in Kusamala+, a multifaceted community‐based intervention. Participation in five activities (home visits by community caregivers, play therapy, church talks, physiotherapy and registration with the Zambian Association of Persons with Disabilities) (that provided cash transfers for families) was associated with change in the three Beach Center Family Quality of Life domains of emotional, physical and disability‐related quality of life. Analysis included descriptive statistics, calculating change in quality of life over time and multinomial linear regression that compared any versus no participation, the number of events and which event was most associated with quality of life change.

**Results:**

All three domains increased, with a statistically significant change in physical and disability‐related quality of life. Nearly all families participated in at least one activity. The most common activity was the community caregiver home visits (87%), followed by assessment by the Zambian Association of Persons with Disability (68%), physiotherapy (49%), play therapy (41%) and church talks (20%). Regression models indicated that physiotherapy was an important predictor of positive change in all three domains, community caregiver visits and play groups were key for emotional quality of life improvement, and Zambian Association of Persons with Disability registration was important for improvement in physical and disability‐related domains. Play groups were also associated with positive change for disability‐related quality of life.

**Conclusion:**

Community‐based interventions that reduce barriers to access and increase social support and physical well‐being are key to improving the quality of life for CLWD and their families in low‐resource settings.

## Summary

- Quality of life for families with a child living with a disability can improve with programmes that increase social support and skills.
- Community‐based services decrease barriers to participation.
- Strong collaborative partnerships are needed to improve the quality of life of families with a child with a disability.

## 1 Background

The quality of life experienced by children living with disabilities (CLWD) and their families is important for both the child and their family to thrive. According to UNICEF (2021), roughly 240 million children under the age of 17 have a disability worldwide. In Eastern and Southern Africa, approximately 28.9 million children under the age of 17 have a disability (UNICEF 2021). According to the most recent Zambia National Disability Survey, completed nearly 10 years ago, the prevalence of disability among children ages 2–17 is estimated at 4.4% (Central Statistical Office, Ministry of Community Development and Social Services 2018). CWLD in Zambia are likely under‐reported considering the most recent statistics found by UNICEF and the tendency to only report individuals with significant disabilities in low‐ and middle‐income countries (Eide and Loeb 2016). Yet, few research studies have considered what interventions are most successful in enhancing outcomes for CLWD and their families (Smythe et al. 2021).

### 1.1 Caring for a CLWD in a Low‐Resource Context

Families of CLWD may experience socio‐economic challenges in caring for their CLWD, including living in poverty, poor access to health and education and stigma. There is a bidirectional relationship between poverty and persons with disabilities. Poverty can contribute to having a child with a disability due to healthcare access, environmental conditions, increased risk of infectious diseases due to living conditions and nutritional deficits (Saeed et al. 2022). Exposure to household poverty early in a child's life can lead to an increased risk of cognitive delays (Victora et al. 2022). Having a child with a disability can increase the risk of poverty for a family because of fewer opportunities for dual‐income families, as fathers often leave families when a childhood disability is evident (Adugna et al. 2020; Mwinbam et al. 2023) or women leave paid work to provide additional care (Heng et al. 2022). Primary caregivers may be unable to leave the child unsupervised to find paid work.

Healthcare services might also be curtailed in this population with the ‘triple A's’ of accessibility, affordability and availability driving limited uptake. Olusanya et al. (2018) noted that healthcare for CLWD is often not easily accessed due to fragmented services often only available in urban areas. Additionally, public transportation to medical care can also be expensive or not equipped to support people with disabilities and buildings might be difficult to navigate, contributing to additional barriers to accessing services (Hashemi et al. 2022). Bright and Kuper's (2018) systematic review identified five studies documenting higher healthcare spending among individuals with disabilities although only three found no difference, leading to their conclusion that financial accessibility remains a barrier to accessing services. Limited access to mobility devices can also lead to difficulties in accessing healthcare as parents struggle to carry children on their backs as they get older (Hashemi et al. 2022).

### 1.2 Family Quality of Life in the Context of Low‐ and Middle‐Income Countries

The changes and challenges that families of CLWD encounter can impact the family's quality of life (FQoL), defined as ‘a dynamic sense of well‐being of the family, collectively and subjectively defined and informed by its members, in which individual and family‐level needs interact’ (Zuna et al. 2010, 73). Considering the complexity of caring for a child living with a disability, this concept captures interactions between services, the individual with a disability and the family (Francisco Mora et al. 2020; Isaacs et al. 2007; Poston et al. 2003; Zuna et al. 2010). This concept has been used to investigate the impact of interventions to support families of CLWD, but the majority of this literature is from high‐income countries (Alnahdi et al. 2022; Jensen‐van Vuuren et al. 2022). Jensen‐van Vuuren et al. (2022) identified 53 articles for inclusion in their scoping review of FQoL in low‐ and middle‐income countries settings, but only one used an FQoL outcome to evaluate the effectiveness of a parent‐training programme in Ghana, with Zuurmond et al. (2018) using a pre–post design that included a measure of family quality of life to build evidence of intervention effectiveness. This study also uses a pre–post design to evaluate the effectiveness of a community‐based intervention to support families and CLWD in Lusaka, Zambia, adding to the limited literature that considers this topic.

Cross‐sectional research on family quality of life demonstrates a relationship between support and this outcome. Jensen‐vanVuuren et al. (2022) considered four categories of support, including financial/material, informal, physical and emotional, which align closely to the literature on coping. In the coping literature, two general categories exist. Problem‐focused coping ties to gaining knowledge of the child's condition, seeking treatment and learning new caregiving skills (McNally and Mannan 2013; Tilahun et al. 2016). Emotion‐focused coping considers acceptance of the child, hope for the future and faith, often associated with a belief that their child is a gift from God (Aldersey 2012; Hepperlen, Rabaey et al. 2021; Maswani‐Mwale et al. 2016).

### 1.3 Study Aims

This study considers the relationship between participating in programme activities or not, the type of activities and the change in quality of life domains. Specifically, we examine participation in any of the five most common intervention activities, the number of activities and which activity was most associated with any observed change in the quality of life domain. These interventions include home visits by community caregivers, play therapy, church talks, physiotherapy and registration with the Zambian Association of Persons with Disability that provides access to cash transfers.

## 2 Methods

### 2.1 Study Design

This programme used a pre–post outcome evaluation. Baseline data were collected for all identified and enrolled families only once in 2019. Staff identified households with a child with a disability by door knocking and referral of those living in population‐dense, low‐resource compounds: Kanyama, Kanyama West, Misisi and Chawama in Lusaka, Zambia. These ‘compounds’ are unplanned and informal communities without access to proper sanitation, paved roads or governmental assistance (Hearst et al. 2020; Hepperlen, Biggs et al. 2021). If there was primary caregiver consent, the family was enrolled in Kusamala+ (*n* = 437 families, 2019) (Hearst et al. 2020). Programme participation was dynamic as families reached goals, moved or otherwise discontinued the programme (*n* = 211 families), and new families were enrolled when identified. Endline data collection (2021) was completed for all families with a child with a disability enrolled in Kusamala+ regardless of when they joined the programme or if they had a baseline survey (*n* = 431 families). There were 228 families who completed baseline data collection in 2019 and follow‐up data collection in 2021. There were no statistical differences in primary caregiver age, sex, relationship or marital status between those who left the programme compared to those who stayed for an endline survey, nor between those who enrolled in Kusamala+ after 2019 and those who had a baseline survey. The survey was read aloud in English, Nyanja or Bemba by multilingual trained Catholic Medical Mission Board staff.

A pilot pre‐ and post‐programme was conducted in two low‐income high‐density compounds in Lusaka, Zambia, between 2017 and 2019. The pilot proved to be a strong indicator of feasibility and acceptability using the community caregivers' approach (Hepperlen, Biggs et al. 2021). During that pilot programming, Kusamala+ provided Training of Trainers (ToT) and curriculum to healthcare providers in two compounds, and they subsequently trained and supervised community volunteers called Community Caregivers. The Community Caregivers were volunteers who had previously worked with Catholic Medical Mission Board on other programme initiatives and were also trained by medical and rehabilitation staff to learn about skills related to stigma, child development, caring for CLWD and more. Community Caregivers had an average caseload of 23 children with about 8.5 house visits per child (Hepperlen, Biggs et al. 2021). They provided home coaching for the parents on basic rehabilitation techniques, positioning, feeding and self‐care strategies, cognitive stimulation, social support and referrals to physiotherapy, other medical or social welfare services and registration with the Zambian Association of Persons with Disability. The Zambian Association of Persons with Disability is a government organization that assesses a person, verifies a disability and provides the person with a Disability Identity Card. This legal document has medical details for emergencies, provides access to social services like cash transfers and provides eligibility for other national benefits. Physiotherapy is physical therapy in the US context. The therapy was provided 1:1 directly with the children and included parental training in range of motion, positioning or other physical mobility to support the child's development. Healthcare providers who supervised the community caregivers offered play therapy sessions in the community for families with and without CLWD. These were in public spaces and visible to the broader community. Community caregivers, physiotherapist and other health facility staff would gather families with and without children with disabilities for a series of activities, including singing songs, playing with balls and using home‐based equipment as toys (pots, pans, cups and bowls). Families were exposed to ways of supporting their child in playtime activities and appropriate positioning. Children without disabilities interacted and played with children with disabilities in a positive space. Supervisors and Catholic Medical Mission Board staff attended churches and provided talks on the rights of persons with disabilities, emphasizing both understanding disability and removing barriers and stigma. Whereas there were other activities, including cooking demonstrations to provide the proper texture of foods for children with swallowing difficulties and increase the nutritional content of foods, the most commonly encountered activities were home visits, physiotherapy, play groups, church talks and access to the Zambian Association of Persons with Disability.

The Institutional Review Boards at the US and Zambian‐based universities and the National Ministry of Community Development approved the project (HRP‐503). This analysis was approved by the Institutional Review Board at a third US‐based university.

### 2.2 Data

#### 2.2.1 Definition of Variables

This intervention included pre‐ and post‐questionnaires, including three FQoL subscales from the Beach Center Family Quality of Life survey (Hoffman et al. 2006) among families of CLWD from 2019 to 2021. The three scales included emotional well‐being, physical well‐being and disability‐related support. A previous study considered the applicability of these scales, which were developed in the context of a high‐income country and used in this resource‐limited area, finding that these items functioned well (Hepperlen, Rabaey et al. 2021). The physical domain is a five‐item scale that includes statements such as ‘my family members have transportation to get to the places they need to be’ and ‘my family has a way to take care of our expenses’. The emotional domain is four items, including questions such as ‘my family has the support we need to relieve stress’ and ‘my family members have friends or others who provide support’. The disability‐related scale includes four items, such as ‘my family member with special needs has support to make progress at home’ and ‘my family member with special needs has support to make friends’. Response options ranged from 1 = *very dissatisfied* to 5 = *very satisfied*. Coefficient alpha was calculated for each of the domains to assess reliability among the three FQoL subscales: emotional (alpha coefficient = 0.80), physical (alpha coefficient = 0.83) and disability‐related domain (alpha coefficient = 0.88). The team used these outcome measures as they were most salient to the interventions provided in this programme. Basic demographic data were collected from adult guardians by trained Catholic Medical Mission Board staff on areas including caregiver marital status, education and age. Caregivers were also asked additional questions related to social support and stigma. Child demographic characteristics, along with type, cause of disability and functional status, were included. Some parents described their child's disability as sickle cell or unable to sit due to natural weakness. For analysis, those conditions were categorized as physical disability. Both accounted for children with both mental and physical disabilities. Activities included home visits by community caregivers, participating in play therapy, attending church talks on stigma reduction, attending physiotherapy and registration by the Zambian Association of Disability.

Study data were collected and managed using REDCap (Research Electronic Data Capture) electronic data capture tools hosted at a US Midwest university (Harris et al. 2009; Harris et al. 2019). REDCap is a secure, web‐based software platform designed to support data collection for research studies, providing (1) an intuitive interface for validated data capture; (2) audit trails for tracking data manipulation and export procedures; (3) automated export procedures for seamless data downloads to common statistical packages; and (4) procedures for data integration and interoperability with external sources.

#### 2.2.2 Statistical Analysis

The data were analysed using Stata v 15.1 (StataCorp, College Station, TX). Descriptive data, including means and proportions of participant and child characteristics, were reported. Change scores for the three domains were calculated by subtracting baseline from endline domain scores. Total activities were a sum of reported activities and were converted to a binary variable as participation in no activities versus one or more. Unadjusted and adjusted regression models included examining the relationship between the number of activities, attending any activities and activity type and the three domains. Adjusted models included which compound community they lived in, parent age, relationship to the child of the person completing the survey, marital status, child sex, child age and child type of disability (physical, cognitive or both).

## 3 Results

Two hundred twenty‐eight families completed the baseline and endline surveys. See Table 1 for population characteristics from endline surveys. More primary caregivers were recruited and retained in the Chawama compound (*n* = 73%) compared to the other compounds. Primary caregivers were predominantly female (94%); the parents of the child with a disability (86%) were never married, separated, divorced or widowed (71%), had some secondary education or less (75%) and were between the ages of 30 and 49 years (58%). There were slightly more male than female children, the mean child age was early 9 years, and primary caregivers described the disability as mental (16%), physical (43%) or both or other (41%). See Table 1.

**TABLE 1:** Characteristics of families and children living with disabilities (CLWD), Kusamala+ in Lusaka, Zambia, 2019–2021.

	*N = 228*	% of total
Gender of parent		
Male	13	5.8
Female	213	94.2
Relationship to child		
Parent	195	85.5
Grandparent	23	10.1
Other relative	10	4.4
Community location		
Kanyama	28	12.3
Kanyama West	14	6.1
Misisi	19	8.3
Chawama	167	73.3
Child's type of disability		
Mental	36	15.8
Physical	99	43.4
Both or other (*n* = 2)	93	40.8
Gender of child		
Male	125	54.8
Female	103	45.2
Child age, years (mean, SD)	8.7 (5.1)	
Caregiver marital status		
Never married, separated, divorced, widowed	93	29.2
Married	133	70.8
Caregiver education status		
Some secondary or less	169	75
Secondary or higher	49	21.6
Other	8	3.5
Caregiver age		
19–29	49	22.7
30–49	133	58.9
50+	44	19.5
Total number of joined programme activities		
Participated in at least one activity	219	96

At the endline, about 23% (*n* = 51) of the CLWD attended school, whereas 77% (*n* = 175) did not. Of the children who did not attend school, caregivers reported that not attending school was due to their disability (39%, *n* = 69). Other reasons why children did not attend school included financial reasons (30%, *n* = 53), they were too young (19%, *n* = 34), transportation (5%, *n* = 8) and other reasons (6%, *n* = 10).

### 3.1 Examination of Intervention Types

Table 2 provides a summary of the FQoL domain change scores and the distribution of activities. All three domains showed a positive change over time, and statistically significant changes were discovered in the physical and disability‐related domains. On average, participants participated in between two and three unique activities, with 96% participating in at least one. The most common activity was the community caregiver home visits (87%), followed by assessment by the Zambian Association of Persons with Disability (68%), physiotherapy (49%), play therapy (41%) and church talks (20%) (data not shown).

**TABLE 2:** Summary statistics of change in family quality of health domains and participation in key activities, Kusamala+, 2019–2021.

	Mean	Standard deviation	Min	Max
Domain (change)				
Emotional (*n* = 222)	0.13	3.8	−10	8
Physical (*n* = 226)	0.70	4.1	−9	12
Disability‐related support (*n* = 226)	1.58	4.1	−12	12

Table 3 presents unadjusted and adjusted regression models. The number of activities was statistically associated with the change in emotional, physical and disability‐related quality of life. Any activity versus no activity was only significant in relation to the emotional quality of life (coef = 4.75, *p* = 0.001). Community caregiver visit (coef = 1.78, *p* = 0.02), play group participation (coef = 2.01, *p* = 0.00) and physiotherapy (coef = 1.03, *p* = 0.04) were significantly associated with emotional quality of life. Physiotherapy (coef = 1.27, *p* = 0.02) and registration with the Zambian Association of Persons with Disability (coef = 1.94, *p* = 0.003) were statistically associated with the change in physical quality of life. Physiotherapy (coef = 1.14, *p* = 0.04) and the Zambian Association of Persons with Disability (coef = 1.37, *p* = 0.03) were statistically significant in unadjusted models of change regarding the change in the disability‐related quality of life. Adjusted models were attenuated, and the Zambian Association of Persons with Disability enrolment became non‐significant, although play groups and physiotherapy remained statistically related to the change.

**TABLE 3:** Unadjusted and adjusted linear regression results of family quality of life domains by activity type, Kusamala+, 2019–2021.

	Unadjusted			Adjusted^a^		
Independent variables^b^	Coef	SE	*p*	Coef	SE	*p*
Emotional quality of life change						
Number of activities	1.12	0.22	0.00	1.17	0.22	< 0.001
Any activity versus none	4.75	1.45	0.001	4.68	1.46	0.002
Activity type						
Community caregiver visit				1.78	0.78	0.03
Play group				2.01	0.52	0.00
Church talk				0.24	0.61	0.70
Physiotherapy				1.03	0.51	0.04
Zambian Association of Persons with Disability				0.38	0.58	0.51
Physical quality of life change						
Number of activities	0.70	0.24	0.005	0.72	0.24	0.003
Any activity versus none	0.37	1.60	0.98	0.02	1.59	0.99
Activity type						
Community caregiver visit				−0.82	0.87	0.34
Play group				−0.23	0.57	0.69
Church talk				1.01	0.68	0.14
Physiotherapy				1.27	0.56	0.02
Zambian Association of Persons with Disability				1.94	0.64	0.003
Disability‐related quality of life change						
Number of activities	0.93	0.23	< 0.001	0.87	0.24	< 0.001
Any activity versus none	0.62	1.60	0.70	0.53	1.61	0.74
Activity type						
Community caregiver visit				0.32	0.37	0.71
Play group				0.93	1.60	0.11
Church talk				−0.14	0.68	0.83
Physiotherapy				1.14	0.57	0.04
Zambian Association of Persons with Disability				1.37	0.65	0.03

^a^ Adjusted models include type of disability, compound, parent age and relationship to child, marital status, child age and child sex.

^b^ Models of total, any and by type of activity were run independently.

## 4 Discussion

The purpose of this analysis was to assess the effects of participating in programme activities or not and to determine which interventions had the greatest impact on family quality of life for each domain of emotional well‐being, physical well‐being and disability‐related support. A change in the emotional quality of life domain was observed if families participated in any activity and were most influenced by the community caregiver visit, play group participation and physiotherapy, whereas the physical quality of life domain change was influenced by physiotherapy and the Zambian Association of Persons with Disability registration. Disability quality of life change had the same influences of participation as the physical quality of life along with play groups and indirectly registration with the Zambian Association of Persons with Disabilities.

In each of these interventions, social support was inherent in the activities. Play groups were organized in central locations in the community. Physiotherapy attended the play groups and provided training for the community caregivers. There is compelling evidence that play groups provided many benefits to parents, including reducing their isolation, feelings of self‐stigma and increased social support (Hepperlan, Rabaey et al. 2021). An evaluation of the pilot Kusamala+ programme indicated that the church talks were instrumental in reducing stigma and discrimination (Hepperlen, Biggs, et al. 2021). It is not clear why the church talks were less impactful for change in quality of life. In Zambia, the COVID‐19 ‘lockdown’ began in March of 2020, although fairly short‐lived and not well enforced, included the banning of religious gatherings. All programme activities complied with the COVID‐19 restrictions. The response in Zambia aimed at reducing super‐spreader events (weddings) but could not restrict all public gatherings. Instead, the government and international organizations emphasized personal protective measures, hand washing and sanitizing and taking temperatures (United Nations 2021). While there were some economic consequences, the cases and mortality due to COVID‐19 were low (Carcelen 2022), although the pandemic did have a bigger impact on families with CLWD (Hearst 2021).

The physical quality of life domain change was influenced by physiotherapy and the Zambian Association of Persons with Disability registration. Physiotherapy was key to improving functional skills for the children, and the cash transfers supported physical well‐being, such as more secure housing and food access. The Kusamala+ activities were largely conducted in the community. The Zambian Association of Persons with Disability registration used to require families to travel with their child to their office. An important change for Kusamala+ was that the Zambian Association of Persons with Disabilities set up a table in the community setting to conduct their assessments and issue the formal documentation. This act substantially reduced barriers for families to access the registration. Physiotherapy was key to improving functional skills for the children, and the cash transfers supported physical well‐being, such as more secure housing and food access.

Change in disability‐related quality of life was associated with play groups, physiotherapy, and the Zambian Association of Persons with Disability. This is also expected as physiotherapy and play groups were directed at improving functional skills for CLWD. The cash transfers likely supported other elements of life, including access to adaptive equipment.

The delivery of activities directly relates to the impact and effectiveness of interventions, particularly regarding FQoL support. Group‐based interventions such as caregiver empowerment sessions and social support groups are effective methods in improving parental resilience and FQoL of parents and CLWD (Bunning et al. 2020; Lu et al. 2018). The interventions implemented for Kusamala+ incorporated elements of social support, such as home visits, play groups and the Zambian Association of Persons with Disability, which has improved emotional, physical and disability‐related support domains.

Home visiting programmes are instrumental in promoting child health outcomes. Community health caregiver‐driven interventions are not only cost‐effective but have a greater ability to reach more families that tend to be unreached (Arasu and Shanbhag 2023). Community caregivers' home visits, play groups and physiotherapy interventions have a greater impact on the emotional domain. The timing of interventions also plays a role in the health outcomes. Early intervention programmes are associated with higher maternal competence, helpfulness and positive family relationships (Crossman et al. 2018).

A similar study evaluating the effectiveness of community health caregiver intervention in rural Karnataka, India, found an increase in the physical domain score (Arasu and Shanbhag 2023). Of the interventions implemented in Kusamala+, physiotherapy and the Zambian Association of Persons with Disability had the greatest influence on the physical domain, as the interventions improved the child's functional skills and connection to services that assist with providing secure housing and nutrition.

### 4.1 Strengths and Limitations

A strength of this study was that the interventions included all disability types and ages of CLWD. With an inclusive criterion for participation, many families were able to access resources and supportive programmes aimed at improving their FQoL. With around 96% of families participating in at least one intervention, this indicates the need and families' willingness to participate and seek support. Parenting is a lifetime role; however, caregiving for a child with disabilities can require additional support beyond childhood. This inclusive criterion demonstrated the need for support regardless of age and disability severity.

Another strength of these interventions was the ability to build capacity for community networks, resources and support. All of the interventions that were implemented had elements of social support that were family‐centred and community‐based, such as the community caregiver and physiology visits. Through this, families were able to build connections with each other, community caregivers and health professionals that led to improvements in the emotional, physical and disability‐related support domains of the FQoL, with statistically significant increases in the physical and disability‐related support domains.

Although the programmes had some benefits, there were several limitations that impacted the effectiveness of these programmes. One limitation of this study was a loss of follow‐up. COVID‐19 was disruptive globally and certainly impacted family housing stability (Hearst et al. 2021). As a result, loss of follow‐up occurred due to participants moving out of the area, leaving behind no contact information. Access to technology such as cell phones and computers in rural areas is slim to none; therefore, follow‐up methods are limited to door‐to‐door surveillance and word of mouth. With the pandemic in full effect, fear of the virus could have potentially discouraged families from continuing to seek and maintain health visits (Zar et al. 2020).

In addition, the sample size for each of the intervention groups varied greatly. The intervention activities provided families with close connections to the health facilities through home visiting community health visits, play groups, church talks, physiology visits at the clinic and social support referrals. The sample size among the intervention groups varied, which may have impacted the overall evaluation of intervention impact. Despite the intervention sample population, participation in at least one of the interventions had improved the family quality of life in all of the domains. Per domain, the large standard deviations indicate a wider dispersion, indicating that there is large variability around the mean. It is likely that some families did substantially better and some did substantially worse for each domain without clear reasons.

Lastly, there were a fair number of families that were not receiving Zambian Association of Persons with Disabilities benefits. One factor that may have impacted the family registration may have been due to the COVID‐19 pandemic. To ease the process of obtaining the Zambian Association of Persons with Disabilities benefits, the government had set up a table in the community to conduct their assessments and issue the formal documentation. However, with the pandemic, many face‐to‐face services were paused and/or delayed worldwide. In addition, it was noted that the Zambian Association of Persons with Disabilities had not increased its capacity to support the families in need, resulting in extended wait times and delays (Hepperlan, Rabaey et al. 2021). However, for the families that did receive this benefit, they experienced an improvement in the physical and disability‐related domains, indicating that the benefit improves the quality of life in those domains.

## 5 Conclusion

This analysis was aimed at assessing participation in programme activities that had the greatest impact on family quality of life in the emotional, physical and disability‐related support domains. Each of the interventions had an impact on the domain changes. Participation in physiotherapy, play groups and community caregiving interventions influenced change in the emotional, physical and disability‐related support domains. This intervention improved all of the domains likely because community caregivers provided much‐needed resources and connections that involved supporting families with resources to assist with expenses, relieving stress and providing support to family members with special needs. As for the physical and disability‐related support domains, the physiotherapy and the Zambian Association of Persons with Disabilities interventions had the greatest impacts as they were connected to governmental support that allows families to receive supplemental care for their loved ones. The interventions demonstrated the need for strong collaborative partnerships and community‐based interventions aimed at supporting families of CLWD. Ideally, government policies should specifically earmark community funds to support and provide a safety net for these families. All children and families deserve to live their optimal lives and be able to fully participate in civic and social life.

## Authorship

MG led the manuscript, drafted the manuscript, conducted preliminary analysis; RH mentored the analysis and edited; JB edited the manuscript; PR edited the manuscript; MH finalized and revised the manuscript and analysis, edited, supervised MG.

## Author Contributions

**Mezan Ghebre:** formal analysis (equal), writing – original draft (equal), writing – review and editing (equal). **Renee Hepperlen:** writing – review and editing (equal). **Jennifer Biggs:** writing – review and editing (equal). **Edgar Lunda:** investigation, supervision, writing – review and editing (equal). **Watson Mwandileya:** investigation, supervision, writing – review and editing (equal). **Paula Rabaey:** conceptualization, writing – review and editing (equal). **Mary O. Hearst:** conceptualization (lead), formal analysis (equal), funding Acquisition, methodology, project administration, supervision, writing – review and editing (equal).

## Funding

This project was funded by the GHR Foundation.

## Ethics Statement

This research was approved as exempt by the University of St. Catherine, University of St. Thomas, and University of Zambia Institutional Review Boards.

## Conflicts of Interest

The authors declare no conflicts of interest.

## Data Availability

Data available upon request from the author.
